# Phylogenetic Characterizations of Highly Mutated EV-B106 Recombinants Showing Extensive Genetic Exchanges with Other EV-B in Xinjiang, China

**DOI:** 10.1038/srep43080

**Published:** 2017-02-23

**Authors:** Yang Song, Yong Zhang, Qin Fan, Hui Cui, Dongmei Yan, Shuangli Zhu, Haishu Tang, Qiang Sun, Dongyan Wang, Wenbo Xu

**Affiliations:** 1 WHO WPRO Regional Polio Reference Laboratory and Ministry of Health Key Laboratory for Medical Virology, National Institute for Viral Disease Control and Prevention, Chinese Center for Disease Control and Prevention, Beijing, People’s Republic of China; 2 Xinjiang Uygur Autonomous Region Center for Disease Control and Prevention, Urumqi City, Xinjiang Uygur Autonomous Region, People’s Republic of China

## Abstract

Human enterovirus B106 (EV-B106) is a new member of the enterovirus B species. To date, only three nucleotide sequences of EV-B106 have been published, and only one full-length genome sequence (the Yunnan strain 148/YN/CHN/12) is available in the GenBank database. In this study, we conducted phylogenetic characterisation of four EV-B106 strains isolated in Xinjiang, China. Pairwise comparisons of the nucleotide sequences and the deduced amino acid sequences revealed that the four Xinjiang EV-B106 strains had only 80.5–80.8% nucleotide identity and 95.4–97.3% amino acid identity with the Yunnan EV-B106 strain, indicating high mutagenicity. Similarity plots and bootscanning analyses revealed that frequent intertypic recombination occurred in all four Xinjiang EV-B106 strains in the non-structural region. These four strains may share a donor sequence with the EV-B85 strain, which circulated in Xinjiang in 2011, indicating extensive genetic exchanges between these strains. All Xinjiang EV-B106 strains were temperature-sensitive. An antibody seroprevalence study against EV-B106 in two Xinjiang prefectures also showed low titres of neutralizing antibodies, suggesting limited exposure and transmission in the population. This study contributes the whole genome sequences of EV-B106 to the GenBank database and provides valuable information regarding the molecular epidemiology of EV-B106 in China.

The genus *Enterovirus*, within family *Picornaviridae*, order *Picornavirales*, consists of 12 species: enterovirus A–H, J, and rhinovirus A–C (www.picornaviridae.com). Based on genomic characteristics, human enteroviruses (EV) can be divided into four species: EV-A, EV-B, EV-C, and EV-D, including more than 100 serotypes of polioviruses (PVs), Coxsackie A viruses (CVAs), Coxsackie B viruses (CVBs), echoviruses, and enteroviruses named with digital serial numbers since EV-D68 was designated^1^,^2^,^3^,^4^. Human enteroviruses are small, non-enveloped, single-stranded, and positive-sense RNA viruses. The genome, composed of approximately 7500 nucleotides (nt), contains a long open reading frame (ORF) flanked by a 5′-untranslated region (UTR) and a 3′-UTR. The ORF can be translated into a 2200-amino acid-long polyprotein, and then cleaved into the 3 polyprotein precursors P1, P2, and P3, which encode the structural proteins VP4, VP2, VP3, and VP1, non-structural proteins 2A, 2B, and 2C, and non-structural proteins 3A, 3B, 3C, and 3D. The mature proteins and precursors, such as 2BC, 3AB, 3CD, participate in replication. The 5′-UTR is roughly 740-nucleotides in length and includes a structure known as the internal ribosome entry site, which is vital for the initiation of translation. The 3′-UTR, consisting of approximately 100 nucleotides containing a long poly (A) stretch, is important in RNA replication^5^,^6^.

EVs can cause a wide range of diseases such as acute flaccid paralysis (AFP), acute aseptic meningitis, encephalitis, myocarditis, hand-foot-and-mouth disease, and others. However, most EV infections are asymptomatic or lead to only mild symptoms resembling the common cold or slight febrile diseases. Classification of EVs is now based on variations in the *VP1* coding region containing a serotype-specific sequence and is known as the “molecular serotyping” method. In this method, strains possessing more than 75% *VP1* nucleotide similarity (85% amino acids similarity) are considered to belong to the same serotype. In contrast, strains with less than 70% *VP1* nucleotide similarity are considered to belong to different serotypes. *VP1* nucleotide similarity between 70–75% is considered the “grey zone”, for which additional information such as neutralization testing with specific antiserum or sequences of other regions to assist with the identification is required^2^,^7^,^8^,^9^,^10^. Because the “molecular serotyping” method has gradually replaced the traditional labour-intensive and time-consuming neutralization test method, it is now the major EV serotyping criterion used in the laboratory.

Enterovirus B106 (EV-B106) is a newly identified serotype within EV-B. The prototype strain (BAN2001-10634) was isolated in Bangladesh in 2001; however, sequences of the prototype strain were not released in the GenBank database. To date, there is only one EV-B106 full-length genome sequence in the GenBank database (strain 12148/YN/CHN/12, hereafter referred to as strain 12148/YN), isolated from an AFP patient in Yunnan Province, China in 2012^11^. Apart from that, only one complete *P1* sequence of EV-B106 (strain PAK_NIH_SP1202B, hereafter referred to as strain SP1202B) from Pakistan in 2010^12^ and a partial *VP1* sequence of EV-B106 (strain BOL/03-10665A, hereafter referred to as strain 10665A) from Bolivia in 2003^13^ were deposited in the GenBank database. In this study, we determined the full-length genome sequences of four EV-B106 strains (strain HTPS-QDH11F/XJ/CHN/2011, strain KS-KSH28F/XJ/CHN/2011, strain KS-MGTH90F/XJ/CHN/2011, and strain AKS-AWT-AFP2F/XJ/CHN/2011, hereafter referred to as HTPS-QDH11F, KS-KSH28F/XJ, KS-MGTH90F, and AKS-AWT-AFP2F, respectively) isolated in Xinjiang Uygur Autonomous Region of China in 2011. These strains were characterised by phylogenetic analysis, antigenic analysis, and seroepidemiology survey.

## Results

### Isolation and molecular typing of the four Xinjiang strains

All four viral strains (HTPS-QDH11F, KS-KSH28F/XJ, KS-MGTH90F, and AKS-AWT-AFP2F) were isolated from human rhabdomyosarcoma (RD) cells. After a complete EV-like cytopathic effect (CPE) was observed, the cell cultures were harvested. The four strains were confirmed to belong to EV-B when the *VP4* sequences were amplified using the species-specific primer pairs EVP4 and OL68-1 and analysed using an online enterovirus genotyping tool^14^,^15^. The entire *VP1* sequences of these four EV strains were analysed using the Basic Local Alignment Search Tool (BLAST) server by comparison with sequences available in the GenBank database; EV serotypes were determined using the online enterovirus genotyping tool^15^. The results indicated that the *VP1* sequences of the four Xinjiang strains showed the highest similarity with those of the EV-B106 strain in the GenBank database, which reached a percentage of as high as 92.8%; therefore, based on the EV molecular typing criteria, the four strains were identified as EV-B106.

### Full-length genomic analysis of the four Xinjiang EV-B106 strains

The full-length genome sequences of the four Xinjiang EV-B106 strains were determined. The results showed that they were all 7421–7422 nucleotides in length with a single ORF of 6579 nucleotides encoding a single polypeptide of 2192 amino acids. The sequences were flanked by a non-coding 5′-UTR of 741–742 nucleotides and non-coding 3′-UTR of 101 nucleotides, with the latter preceding a long poly(A) tail. The overall compositions of the four stains (HTPS-QDH11F, KS-KSH28F/XJ, KS-MGTH90F, and AKS-AWT-AFP2F) were 28.39–28.68% A, 23.73–24.01% C, 24.43–24.70% G, and 22.93–23.16% T, respectively. Because the full-length sequence of the prototype strain of EV-B106 was unavailable, we aligned the full-length genomes of the four Xinjiang strains with the Yunnan strain (12148/YN). The results showed that all four Xinjiang EV-B106 strains contained three nucleotide insertions at positions 7318, 7332, and 7425 and had one or two deletions compared to the Yunnan strain (12148/YN). Strain HTPS-QDH11F had two deletions at positions 95 and 7317; strain KS-KSH28F/XJ had one deletion at position 7317; strain KS-MGTH90F had two deletions at positions 95 and 7347; and strain AKS-AWT-AFP2F had one deletion at position 7347.

Pairwise comparisons of the nucleotide sequences and the deduced amino acid sequences were conducted among the four Xinjiang EV-B106 strains with the Yunnan EV-B106 strain and Pakistan EV-B106 strain, as well as of other prototype strains with the EV-B species (Table 1). The complete genome nucleotide sequence and amino acids similarities among the four Xinjiang strains were 93.3–98.1% and 98.7–99.2%, respectively. Furthermore, the four Xinjiang strains showed 80.5–80.8% nucleotide identity and 95.4–97.3% amino acid identity with the Yunnan EV-B106 strain. For the *P1* coding sequences of the four Xinjiang strains, 91.6–92.4% nucleotide identity and 96.9–97.5% amino acid identity were observed when compared with the Pakistan strain.

### Phylogenetic analysis of Xinjiang EV-B106 strains with other EV-B genomes

**Figure 1:**
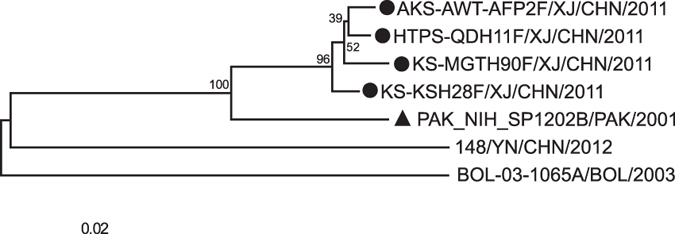
Phylogenetic relationships based on the partial *VP1* region sequences of EV-B106. Four Xinjiang EV-B106 strains described in this study (indicated by circles) and other EV-B106 strains (available in the GenBank database) were analysed from the 303 nucleotides (nucleotide 2585–2887) in the partial *VP1* coding region sequence. The Pakistan strain is indicated with a triangle.

**Figure 2:**
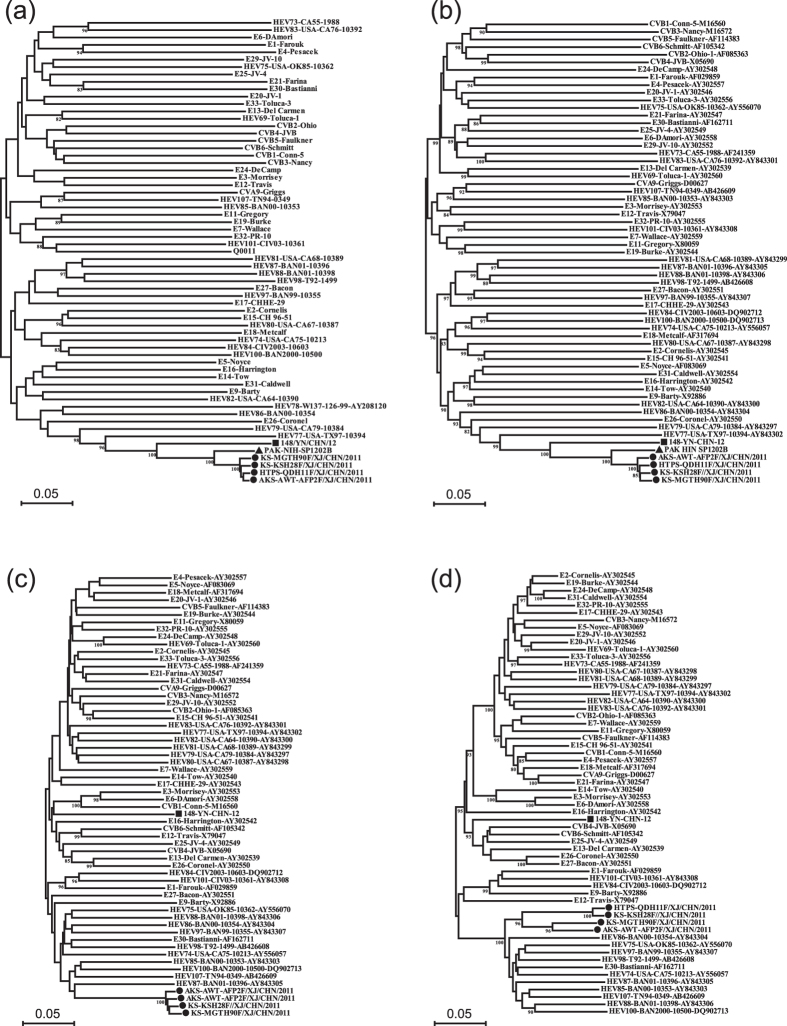
Phylogenetic relationships based on the *VP1, P1, P2*, and *P3* region sequences of EV-B. Four Xinjiang EV-B106 strains (indicated by circles) and 56 other EV-B prototype strains were analysed by nucleotide sequence alignment using the neighbour-joining algorithms implemented in the MEGA 5.0 program. Numbers at the nodes indicate bootstrap support for that node (percentage of 1000 bootstrap replicates). The triangle and square indicates the Pakistan EV-B106 strain and Yunnan EV-B106, respectively. The scale bars represent the genetic distance. All panels have the same scale. (**a**) *VP1* coding sequences; (**b**) *P1* coding sequences; (**c**) *P2* coding sequences; and (**d**) *P3* coding sequences.

A phylogenetic tree was constructed based on the partial *VP1, P1, P2*, and *P3* coding region nucleotide sequences of the four Xinjiang EV-B106 strains, Yunnan EV-B106 strain, Pakistan EV-B106 strain (*VP1* and *P1* region sequences only), Bolivia EV-B106 strain (partial *VP1* region sequence only), and other prototype strains of EV-B in the GenBank database (Figs 1 and 2). Based on the phylogenetic trees constructed from the 303-nucleotide (nt 2585–2887) partial *VP1* sequences of the four Xinjiang strains, all Xinjiang strains clustered with the Pakistan strain and showed significant genetic similarity to the Bolivia strain, but did not cluster with the Yunnan strain (Fig. 1).

As shown in Fig. 2, the phylogenetic trees based on the *P1* coding region sequences revealed that the four Xinjiang EV-B106 strains clustered together with the Pakistan strain and the Yunnan strain, demonstrating that these strains belong to the same serotype EV-B106 and confirming the preliminary molecular typing results. The nucleotide identities of the *VP1* region between the Xinjiang strains (HTPS-QDH11F, KS-KSH28F/XJ, KS-MGTH90F, and AKS-AWT-AFP2F) and Pakistan strain were 92.8%, 92.1%, 91.4%, and 92.2%, respectively. Based on the phylogenetic tree, the EV-B77 prototype strain (strain USA-TX97-10394) showed the highest homology with all EV-B106 strains with a bootstrap support value of 100%. The nucleotide identities of the *VP1* region between the four Xinjiang strains and EV-B77 prototype strain reached percentages as high as 69.5%, 70.2%, 69.1%, and 69.9%, respectively.

Unexpectedly, the four Xinjiang EV-B106 strains did not form a lineage with the only Yunnan EV-B106 strain in the trees based on the *P2* and *P3* coding regions, which suggesting that recombination between EV-B106 and other EV-B serotypes occurred. In the *P2* coding region, the four Xinjiang EV-B106 strains clustered together and shared the highest similarity with the prototype strain EV-B87. In the *P3* coding region, however, even the four Xinjiang strains did not cluster together, and the sequence similarities among their *P3* regions varied widely from 82.9% (strain KS-KSH28F/XJ and KS-MGTH90F) to 97.8% (strain KS-KSH28F/XJ and HTPS-QDH11F). This unexpected finding suggested that the occurrence of recombination events among the four strains were different.

### EV-B106 strains underwent extensive genetic exchanges with other EV-B strains in Xinjiang

**Figure 3:**
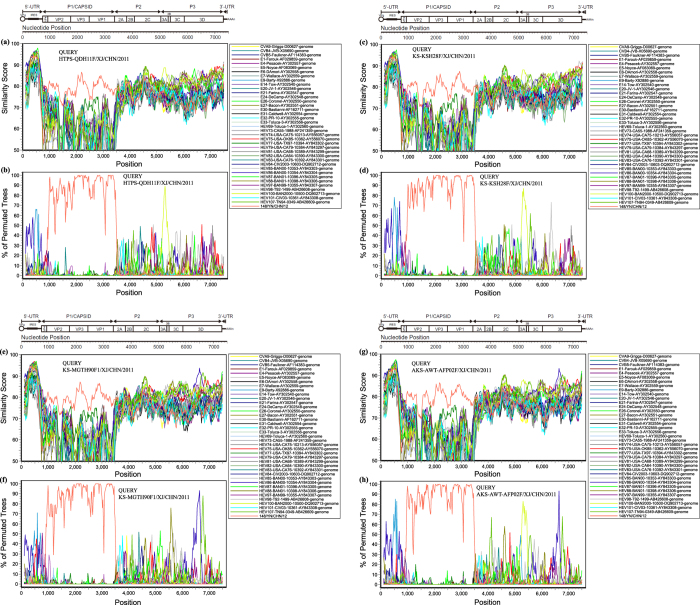
Similarity plots and bootscanning analyses of the whole genomes of Xinjiang EV-B106 strains. (**a**), (**c**), (**e**), (**g**) Similarity plots and (**b**), (**d**), (**f**), (**h**) bootscanning analyses. A sliding window of 200 nucleotides was used, moving in 20-nucleotide steps. The four Xinjiang strains were used as query sequences independently (indicated in the upper right corner of the image).

To confirm the recombination events between the Xinjiang EV-B106 strains and other EV-B strains, similarity plots and bootscanning analyses were performed (Fig. 3). The four strains (HTPS-QDH11F, KS-KSH28F/XJ, KS-MGTH90F, and AKS-AWT-AFP2F) were picked as query sequences. The Yunnan EV-B106 strain was included in these analyses because it was the only available full-length sequence of EV-B106 in the GenBank database.

In the *P1* coding region, the four Xinjiang strains showed the highest similarity with the Yunnan EV-B106 strain (12148/YN), as expected. However, in the other regions, the results revealed that the four Xinjiang strains clearly differed from the Yunnan EV-B106 strain. These results suggest that recombination events occurred in all four Xinjiang EV-B106 strains in the non-capsid regions. In the *P2* region, no obvious donor sequence could be identified. In the *P3* region, however, the four Xinjiang strains all recombined with other EV-B strains, but the donor sequences presented dissimilarities. One recombination event between three Xinjiang strains (excluding strain KS-MGTH90F) and EV-B100 prototype strain was identified from the 3′ end of the *3A* region to the 5′ end of the *3C* region. Bootscanning analysis also supported that strain KS-MGTH90F shared the highest similarity with EV-B86 followed by EV-B107 prototype strains in the *3D* region, confirming the occurrence of recombination. Furthermore, recombination of a small fragment in the 5′-UTR region between strain AKS-AWT-AFP2F and Echovirus 9 prototype strain was also detected.

We further evaluated the recombinant structure of the four Xinjiang EV-B106 strains because of their distinct *P3* region sequences. We screened the *P3* region sequences of the four Xinjiang strains by BLAST, and found that two strains, HTPS-QDH11F and KS-KSH28F, shared the highest nucleotides similarity in the *P3* region with the EV-B85 strain, which was also isolated from Xinjiang, China^16^. This outcome was unexpected because a previous study demonstrated that this Xinjiang EV-B85 strain contained an unknown serotype EV-B donor sequence. Because the serotype EV-B106 had not been identified at that time, we assumed that these four Xinjiang EV-B106 strains contained the donor sequence shared with the EV-B85 strain HYTY-ARL-AFP02F. We then conducted detailed analysis of the four Xinjiang EV-B106 strains with the Xinjiang EV-B85 strain with the *P3* region sequences and observed that strains HTPS-QDH11F and KS-KSH28F/XJ showed 89.9% similarity with the EV-B85 strain HYTY-ARL-AFP02F in the *3D* region.

**Figure 4:**
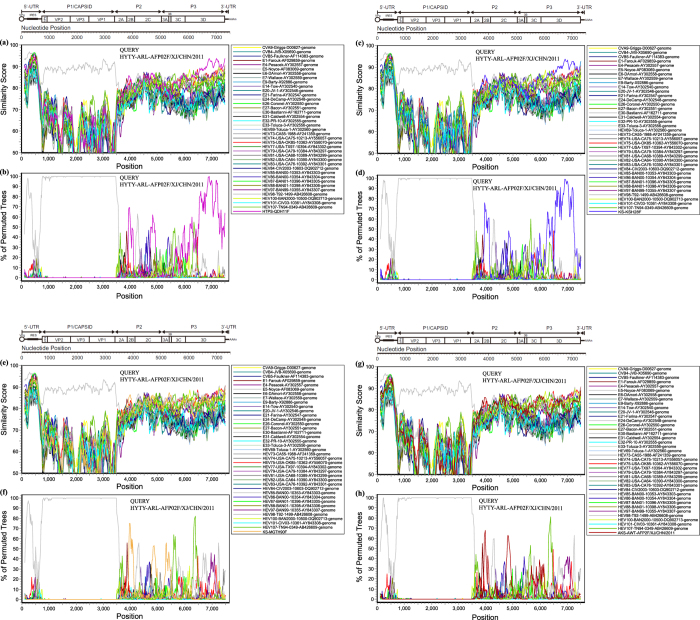
Recombination analyses of the four Xinjiang EV-B106 strains with Xinjiang EV-B85 strain HYTY-ARL-AFP02F. (**a**), (**c**), (**e**), (**g**) Similarity plots and (**b**), (**d**), (**f**), (**h**) bootscanning analyses. A sliding window of 200 nucleotides was used, moving in 20-nucleotide steps. The Xinjiang EV-B85 strain HYTY-ARL-AFP02F was used as a query sequence (indicated in the upper right corner of the image).

To confirm that recombination events between the Xinjiang EV-B106 strains and Xinjiang EV-B85 strain occurred, we conducted both similarity plots and bootscanning analyses on the four Xinjiang EV-B106 strains and Xinjiang EV-B85 strain HYTY-ARL-AFP02F along with other EV-B prototype strains (Fig. 4) using the Xinjiang EV-B85 strain as a query sequence. The results revealed that recombination between the Xinjiang EV-B85 strain HYTY-ARL-AFP02F and the Xinjiang EV-B106 strains (strain HTPS-QDH11F and strain KS-KSH28F/XJ) in the *3D* region were highly possible. The results also confirmed that the EV-B106 strains underwent extensive genetic exchanges with the other EV-B strains, including EV-B85 strains in Xinjiang, and that the unknown serotype EV-B donor sequence for the Xinjiang EV-B85 strain was also present in these Xinjiang EV-B106 strains.

### Xinjiang EV-B106 shows temperature sensitivity

**Figure 5:**
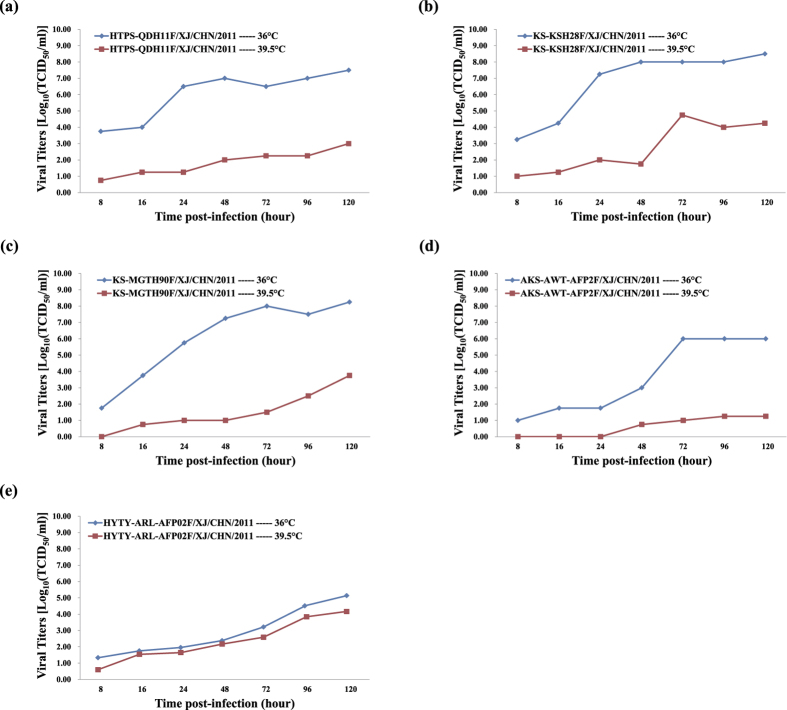
Temperature sensitivity test curves of the four Xinjiang EV-B106 isolates. A Xinjiang EV-B85 strain (strain HYTY-ARL-AFP02F, showing non-temperature sensitivity) was used as an experiment control. The blue and red lines represent the growth trend of the viruses on RD cells at 36 °C and 39.5 °C, respectively. (**a**) strain HTPS-QDH11F (EV-B106); (**b**) strain KS-KSH28F/XJ (EV-B106); (**c**) strain KS-MGTH90F (EV-B106); (**d**) strain AKS-AWT-AFP2F (EV-B106); (**e**) strain HYTY-ARL-AFP02F (EV-B85).

The four Xinjiang EV-B106 strains were compared based on replication capacity at an elevated temperature (39.5 °C). A Xinjiang EV-B85 strain (strain HYTY-ARL-AFP02F, a non-temperature sensitive strain) was used for an experiment control. The results showed that all EV-B106 strains were temperature-sensitive with titre reductions of more than 2 logarithms at 36 °C/39.5 °C (Fig. 5).

### Seroprevalence of EV-B106 in Xinjiang

A total of 50 serum samples were collected from newborns to 4-year-old children from Xinjiang, with 25 were collected from Kashgar prefecture and 25 from Hotan prefecture. From the samples surveyed, 21 were seropositive for EV-B106 (>1:8), with a total positive rate of 42%. The geometric mean titre (GMT) among the seropositive samples was 1:21.5. The composition ratios for the EV-B106 neutralization antibody titres of <1:8, 1:8–1:64, and >1:64 were 58%, 36%, and 6%, respectively. This suggests that compared with the seroepidemiology studies of other widely endemic EVs in China, such as EV-A71 and CV-A16, the positive rate and GMT of EV-B106 was lower^17^; however, the prevalence of EV-B106 was greater than that of other novel EVs isolated in Xinjiang, such as EV-B81 and EV-A89^4^,^18^.

There were some differences between samples collected from the Kashar and Hotan prefectures in regards to the seroprevalence rates and GMTs. In the Kashar prefecture, the positive rate of EV-B106 neutralization antibody and GMTs were 44% and 1:12.4, respectively, while in the Hotan prefecture, they were 40% and 1:39.4 respectively. Although there was no significant difference between the positive seroprevalence rates of the two regions, the GMT in the Kashar prefecture was significantly higher than that in the Hotan prefecture (seroprevalence rate: χ^2^ = 0.082, *p* > 0.05, GMTs: *p* < 0.01).

## Discussion

The prototype strain of EV-B106 was isolated in Bangladesh in 2001, but no sequences were available in the GenBank database until recently. Approximately a decade later, three EV-B106 sequences were reported in the GenBank database from Pakistan, the Yunnan Province of China, and Bolivia in 2010, 2012, and 2013, respectively, and only one full-length genome sequence is available to date^11^,^12^,^13^. Interestingly, the three reported EV-B106 strains were all isolated from AFP patients, indicating possible connections between EVB-106 infections and AFP. However, few studies have evaluated EV-B106 in China or worldwide, and thus additional research is needed to elucidate the possible correlation.

In our study, four Xinjiang EV-B106 strains were described. These EV-B106 strains were isolated in the Hotan, Kashar, and Aksu prefectures, which are all located in the Southwest region of Xinjiang. Compared with the Yunnan EV-B106 strain, the first and only EV-B106 strain previously reported in China, the Xinjiang EV-B106 strains display significantly different characteristics despite belonging to the same EV serotype. Based on the phylogenetic trees, the four Xinjiang EV-B106 strains clustered with the Yunnan strain only in the *VP1* and *P1* coding regions, while forming different lineages in both the *P2* and *P3* coding regions.

Recombination is a well-known phenomenon for enteroviruses^19^,^20^,^21^,^22^,^23^. In our study, similarity plots and bootscanning analyses for the Xinjiang EV-B106 strains were performed to investigate intertypic recombination between EV-B86, EV-B100, and EV-B107 with the *P3* non-capsid region and Echovirus 9 5′-UTR. Additionally, the *P3* region sequences of the four strains were diverse, indicating that EV-B106 had been circulating in the environment for an extended time before isolation^24^. As EV-B86, EV-B100, and EV-B107 are all newly discovered recombinant EVs, this indicates that recombination is a driving force in the formation of novel EV serotypes.

Furthermore, a previously reported Xinjiang EV-B85 strain (strain HYTY-ARL-AFP02F, a representative strain of Xinjiang EV-B85 isolated from an AFP patient) showing high nucleotide similarity with the Xinjiang EV-B106 strains in the *P3* region was screened. A previous study of strain HYTY-ARL-AFP02F indicates that recombination occurred with an unknown EV-B donor sequence^16^. Interestingly, a similar donor sequence was found in two Xinjiang EV-B106 strains, HTPS-QDH11F and KS-KSH28F. Moreover, the EV-B85 strains analysed in the previous study and four EV-B106 strains analysed in this study were all isolated from AFP patients and their healthy contacts in the same region (Southern Xinjiang) in the same year (2011). These conditions are favourable for recombination because recombination between different EV serotypes typically occurs when distinct viruses infect and replicate in the same cell^25^,^26^. Although recombination between EV-B85 and EV-B106 was possible, it is difficult to determine whether the EV-B106 strain was exact the “unknown serotype” EV-B donor for the Xinjiang EV-B85 strains. However, we predict that Xinjiang EV-B106 strains and EV-B85 strains co-circulated with other EV-Bs and underwent extensive genetic exchanges with other EV-B strains; thus, it is likely that related *P3* sequences of Xinjiang EV-B85 and EV-B106 originated from a common unknown EV-B ancestor through recombination.

The four Xinjiang EV-B106 strains were all temperature-sensitive, suggesting that their virulence is currently not high enough to have strong transmissibility^16^,^27^. However, strain AKS-AWT-AFP2F was isolated from an AFP patient, and thus the virulence of EV-B106 cannot be underestimated. Furthermore, because recombination occurred between the four strains and other EV-B strains, particularly with some non-temperature-insensitive strains such as Xinjiang EV-B85 strains, it is difficult to predict whether EV-B106 will evolve and heighten its virulence in the future.

A seroepidemiological analysis of EV-B106 in the Xinjiang Autonomous Region of China was conducted. The results showed that GMTs were higher in the Kashar prefecture than in the Hotan prefecture (*p* < 0.05), suggesting that these EV-B106 strains widely circulated in the Kashar prefecture, particularly because two of the four EV-B106 strains were isolated in the Kashar prefecture. However, compared to the other prevalent EVs in China, such as EV-A71 and CV-A16^17^,^28^, the seropositive rate and GMT of the EV-B106 neutralization antibody in the two regions of Xinjiang were relatively low, demonstrating that the extent of transmission and exposure of the viral population to the novel EV-B106 serotype were highly limited. The low rate of virus isolation also supports this.

In conclusion, we reported the full-length genome sequences of four EV-B106 strains during AFP surveillance in Xinjiang Uygur Autonomous Region, China. To date, only one EV-B106 strain (Yunnan strain) has been reported worldwide, indicating that this serotype is not widespread in China or in the world. Sequence analysis suggested that these four strains have high genetic diversity compared with the Yunnan EV-B106 strain, intertypic recombination in the non-structural protein region of all the four strains, and extensive genetic exchanges with the other EV-B circulating in Xinjiang. The high divergence among the EV-B106 strains reflects that this virus is not newly emergent, but has circulated in the environment for many years. In addition, the differences among the *P3* region sequences of four Xinjiang EV-B106 strains indicate that they have been circulating separately for some time. This study expands the number of whole genome sequences of EV-B106 in the GenBank database and provides valuable information regarding the molecular epidemiology of EV-B106 in China, which can be utilized to evaluate the association between EV-B106 and EV-B106-related diseases such as AFP.

## Methods

### Sample collection

This study did not involve human participants or human experimentation; the only human materials used were stool samples collected from one AFP patient and three healthy children at the instigation of the Ministry of Health P. R. of China for public health purposes. Written informed consent for the use of their clinical samples was acquired from all individuals involved in this study. This study was approved by the Ethics Review Committee of the National Institute for Viral Disease Control and Prevention (NIVDC), Chinese Center for Disease Control and Prevention, all experimental protocols were approved by NIVDC, and the methods were carried out in accordance with the approved guidelines.

The four EV-B106 strains (strain HTPS-QDH11F, strain KS-KSH28F, strain KS-MGTH90F, and strain AKS-AWT-AFP2F) used in this study were isolated from stool specimens from one AFP patient and three healthy children in Hotan, Kashgar, and Aksu Prefectures of Xinjiang Autonomous Region, China. The samples were collected in 2011 during poliovirus surveillance activities.

In the seroprevalence study of EV-B106 antibodies, 50 healthy children ≤5 years of age were surveyed. Fifty serum samples were collected in 2013 for seroepidemiological analysis of enteroviruses, with informed parental consents, by the Xinjiang Center for Disease Control and Prevention: 25 samples from Hotan and 25 samples from Kashgar, the two regions where the strains were isolated. The same serum samples were used previously for a EV-A89 seroepidemiology study^4^. None of the children had any signs of disease at the time of sample collection.

### Viral isolation

Stool samples from the AFP patient and three healthy children were collected and processed according to standard procedures^29^,^30^. The processed samples were then inoculated into two cell lines for isolation: RD cell and a mouse cell line with the human poliovirus receptor (L20B). Both cell lines were provided by the WHO Global Poliovirus Specialized Laboratory in the US and originally purchased from the American Type Culture Collection (Manassas, VA, USA). Infected cell cultures were harvested after complete CPE was observed.

### Molecular typing

Viral RNA was extracted from the cell culture using a QIAamp Viral RNA Mini Kit (Qiagen, Hilden, Germany). Reverse transcription polymerase chain reaction (RT-PCR) was conducted to amplify the partial *VP1* coding region using the PrimeScript One Step RT-PCR Kit Ver.2 (TaKaRa, Dalian, China) with primer pairs 490 and 492^2^. The PCR products were purified using the QIAquick PCR purification kit (Qiagen, Germany) and sequenced in both directions at least once from each strand using ABI 3130 Genetic Analyser (Applied Biosystems, Foster City, CA, USA). The acquired partial *VP1* sequences were analysed with the BLAST server by comparing with the sequences available in the GenBank database and were determined using the EV Genotyping Tool^15^.

### Primer designing and whole genomic sequencing

The 5′ end of the genome sequence was amplified using the 5′-Full RACE Kit (Takara, Shiga, Japan) according to the manufacturer’s instructions. The 3′ end sequence was obtained by using an oligo-dT primer (primer 7500A)^31^ as the downstream primer for amplification. The primers used for PCR amplification and sequencing for the rest of the genome were based on the primer walking method (Table 2).

### Phylogenetic and recombination analyses

The nucleotide and deduced amino acid sequences of the four Xinjiang EV-B106 strains were compared with those of the prototype EV-B strains by pairwise alignment using the MEGA program (version 5.03)^32^. Phylogenetic trees were constructed by the neighbour-joining method implemented in the MEGA program and Kimura 2-parameter model. Bootstrap values of greater than 80% were considered statistically significant for grouping. The SimPlot program (version 3.5.1) was used for similarity plots and bootscanning analyses, with a 200-nucleotide window moving in 20-nucleotide steps. Bootscanning analyses were run with the neighbour-joining method^33^.

### Assay for temperature sensitivity

Temperature sensitivities of the four Xinjiang EV-B106 strains were assayed on monolayer RD cells in 24-well plates^27^. A Xinjiang EV-B85 strain (strain HYTY-ARL-AFP02F, showing non-temperature sensitivity)^16^ was used for an experiment control in order to ensure that cells and thus overall viral replication were not affected at high temperature in experimental conditions. The 24-well plates were inoculated with 50 μL of undiluted virus stocks and placed in two different CO_2_ incubators: 36 °C as the optimal temperature for virus propagation and 39.5 °C as the supraoptimal temperature. After absorption at 36 °C or 39.5 °C for 1 h, the unabsorbed virus inoculum was removed and 100 μL of maintenance medium was added to each well. The plates were incubated again at 36 °C and 39.5 °C and harvested at 8 time points post-infection (4 h, 8 h, 16 h, 24 h, 48 h, 72 h, 96 h, and 120 h) in succession. The 50% cell culture infectious dose (CCID_50_) was calculated by the end-point dilution method on monolayer RD cells in 96-well plates at 36 °C. Virus isolates showing more than a two-logarithm reduction in titre at different temperatures were considered to be temperature-sensitive^34^.

### Neutralizing test

Neutralizing antibodies against EV-B106 were detected in a neutralization test using the human RD cell line as previously described^17^ with some modifications. Strain AKS-AWT-AFP2F was selected as the attack virus strain in the neutralizing test because the four Xinjiang EV-B106 strains appeared to be antigenically equivalent (VP1 amino acid identity among the four EV-B106 strains was 98.9–99.6%) and showed the highest TCID_50_ value among the four Xinjiang EV-B106 strains. Serum samples were inactivated at 56 °C for 30 min, and sample dilutions from 1:4 to 1:1024 were assayed. The mixture of virus samples (50 μL) with a CCID_50_ of 100 and appropriate serum dilution (50 μL) was then incubated at 36 °C in a CO_2_ incubator. After incubation for 7 days, the highest dilution of serum that protected 50% of the cultures was recorded according to the EV-like CPE. A serum sample was considered positive if the neutralization antibody titre was observed at a dilution of 1:8, and GMT was subsequently calculated.

### Statistical analysis

All statistical analyses were performed using SPSS Statistics software (version 19.0) (SPSS, Inc., Chicago, IL, USA). Chi-square test was used to compare the seroprevalence rates of the viral strains between Kashgar prefecture and Hotan prefecture. Mann-Whitney U test was used to compare the differences in GMTs. Titres below 1:8 were assumed 1:4 for calculation. Differences with an error probability of *p <* 0.05 were considered statistically significant.

### Nucleotide sequence accession numbers

The full-length genomic sequences of the four EV-B106 strains (strain HTPS-QDH11F, strain KS-KSH28F, strain KS-MGTH90F, and strain AKS-AWT-AFP2F) described in this study were deposited in the GenBank database under the accession numbers KX171334-KX171337, respectively.

## Additional Information

**How to cite this article**: Song, Y. *et al*. Phylogenetic Characterizations of Highly Mutated EV-B106 Recombinants Showing Extensive Genetic Exchanges with Other EV-B in Xinjiang, China. *Sci. Rep.* **7**, 43080; doi: 10.1038/srep43080 (2017).

**Publisher's note:** Springer Nature remains neutral with regard to jurisdictional claims in published maps and institutional affiliations.

## Figures and Tables

**Table 1:** Pairwise nucleotide and amino acid sequence identities between the four Xinjiang EV-B106 strains, Pakistan EV-106 strain, Yunnan EV-B106 strain, and other prototype strains of the EV-B species.

Region	Nucleotide identity (%) [Amino acid identity (%)]											
	HTPS-QDH11F			KS-KSH28F			KS-MGTH90F			AKS-AWT-AFP2F		
	Pakistan EV-B106	Yunnan EV-B106 Strain	Prototypes of other EV-B	Pakistan EV-B106	Yunnan EV-B106 Strain	Prototypes of other EV-B	Pakistan EV-B106	Yunnan EV-B106 Strain	Prototypes of other EV-B	Pakistan EV-B106	Yunnan EV-B106 Strain	Prototypes of other EV-B
5′-UTR	N/A	85.9	66.8–91.3	N/A	86.2	66.9–91.6	N/A	86.1	66.4–91.1	N/A	85.8	66.6–91.1
VP4	92.2	79.2	66.1–82.1	91.7	79.2	66.6–82.1	90.8	79.7	64.7–83.0	91.7	79.7	67.6–81.6
	(100.0)	(92.7)	(76.8–95.6)	(100.0)	(92.7)	(76.8–95.6)	(100.0)	(92.7)	(76.8–95.6)	(100.0)	(92.7)	(76.8–95.6)
VP2	93.1	81.4	64.2–73.9	93.1	82.3	64.6–74.6	92.8	81.4	64.3–74.3	93.2	81.9	64.0–74.5
	(99.6)	(97.3)	(71.4–87.0)	(99.2)	(96.9)	(71.4–87.0)	(98.8)	(96.5)	(71.4–87.0)	(98.8)	(96.9)	(71.4–86.6)
VP3	91.2	82.0	62.1–74.6	90.9	92.0	62.3–74.6	90.6	82.1	62.0–75.0	91	82.1	62.3–75.2
	(91.6)	(97.0)	(66.5–87.8)	(91.6)	(97.0)	(65.6–87.8)	(99.7)	(97.0)	(65.2–87.4)	(92.0)	(97.9)	(66.1–87.8)
VP1	92.8	79.8	53.1–69.5	92.1	79.8	53.1–70.2	91.4	79.9	53.0–69.1	92.2	80.0	53.2–69.9
	(96.1)	(91.6)	(52.1–78.4)	(95.4)	(91.6)	(52.1–78.4)	(95.8)	(91.6)	(52.1–78.1)	(95.8)	(92.0)	(52.1–78.4)
2A	N/A	81.3	75.2–82.1	N/A	81.7	75.7–82.2	N/A	80.8	74.8–82.0	N/A	82.2	74.6–82.2
	(94.6)	(90.0–96.6)	(94.6)		(90.0–96.6)	(94.6)		(90.0–96.6)	(94.6)		(90.0–96.6)	
2B	N/A	77.7	74.0–82.1	N/A	76.4	74.0–82.1	N/A	76.7	73.4–81.1	N/A	78.1	74.4–82.1
		(96.9)	(93.9–98.9)		(95.9)	(92.9–98.9)		(95.9)	(92.9–98.9)		(96.9)	(93.9–98.9)
2C	N/A	80.7	78.2–84.4	N/A	80.6	77.9–84.9	N/A	80.4	78.0–84.2	N/A	81.2	78.3–85.5
		(96.3)	(95.1–98.1)		(96.9)	(95.7–98.7)		(95.7)	(94.5–97.5)		(96.9)	(95.7–98.7)
3A	N/A	77.1	75.2–86.8	N/A	78.2	73.7–87.2	N/A	79.0	74.5–85.7	N/A	79.7	73.7–88.0
		(96.6)	(92.1–100.0)		(96.6)	(92.1–100.0)		(95.5)	(89.8–98.8)		(96.6)	(92.1–100.0)
3B	N/A	83.3	74.2–84.8	N/A	83.3	74.2–86.3	N/A	72.7	74.2–87.8	N/A	78.7	72.7–87.8
		(90.9)	(90.9–100.0)		(90.9)	(90.9–100.0)		(90.9)	(90.9–100.0)		(90.9)	(90.9–100.0)
3C	N/A	76.6	75.1–84.3	N/A	76.5	74.8–84.1	N/A	77.4	76.5–86.7	N/A	77.2	75.5–85.4
		(96.5)	(50.2–52.9)		(93.9)	(91.8–98.3)		(96.1)	(93.9–100.0)		(95.0)	(92.8–98.9)
3D	N/A	78.9	77.3–85.4	N/A	79.5	77.9–86.6	N/A	78.8	78.0–86.0	N/A	78.9	77.9–85.3
		(95.6)	(94.5–99.3)		(96.3)	(95.2–99.3)		(95.6)	(95.0–99.3)		(95.8)	(95.2–99.3)
3′-UTR	N/A	84.1	75.0–97.0	N/A	85.1	75.9–97.0	N/A	85.2	76.9–100.0	N/A	85.2	76.9–100.0

**Table 2:** PCR and sequencing primer.

Primer	Nucleotide position(nt)	Primer sequence (5′-3′)	Orientation	Reference
0001S48		GGGGACAAGTTTGTACAAAAAAGCAGGCTTTAAAACAGCTCTGGGGTT	Forward	31
5′ RACE-inner-primer-EV106-504A	483–504	TATCTGCTCCACAGTTAGGATT	Reverse	This study
5′ RACE-outer-primer-EV106-726A	705–726	GAGAGTGTTTAATGCTGTGTAG	Reverse	This study
EVP4	541–560	CTACTTTGGGTGTCCGTGTT	Forward	14
OL68-1	1178–1197	GGTAAYTTCCACCACCANCC	Reverse	14
EV106-1100S	1100–1120	GGCAGAGGATCAACCGACTCA	Forward	This study
EV106-1991S	1991–2012	CACATTCCAACTCGATCCAGGC	Forward	This study
EV106-2412A	2392–2412	TTCGCACTGAGAAGTCATTAC	Reverse	This study
490	2226–2248	TGIGTIYTITGYRTICCITGGAT	Forward	35
491	2883–2902	ATGTAYRTICCICCIGGNGG	Forward	35
492	2953–2934	GGRTTIGTIGWYTGCCA	Reverse	35
493	3641–3622	TCNACIANICCIGGICCYTC	Reverse	35
EV106-3035S	3035–3056	CAGCATGTTCTATGATGGCTGG	Forward	This study
EV106-3917A	3898–3917	CCACAATTACCAAGGCTGAT	Reverse	This study
EV106-3703S	3703–3722	AGGGTGTCGTCGGCTTTGCT	Forward	This study
EV106-4894A	4875–4894	CGGGCAGCATTTCTTGAAGT	Reverse	This study
EV106-4784S	4784–4803	GAGGTTTCACTTTGACATGA	Forward	This study
EV106-6043A	6023–6043	CTGAGGACTGCTGGTTCCTTT	Reverse	This study
EV106-6700S	6700–6719	ACCAGCAAGTTCCCAAACAT	Forward	This study
EV106-6888A	6869–6888	ATCATCCTGAACTGGTCCAA	Reverse	This study
EV106-6586S	6586–6605	GGAGTAAGATACCAGTGATG	Forward	This study
EV106-6995S	6995–7014	CATGACACCAGCAGATAAAG	Forward	This study
7500A		GGGGACCACTTTGTACAAGAAAGCTGGG(T)_24_	Reverse	31
